# Assessment of the Effects of Garlic (*Allium sativum* L.) Stalk Incorporation on Soil Fertility and Bacterial Biodiversity

**DOI:** 10.3390/plants14050672

**Published:** 2025-02-21

**Authors:** Fan Huang, Chunmei Wang, Sajjad Raza, Guangfeng Yao, Lihua Xue, Yinku Liang, Xiaoning Zhao

**Affiliations:** 1 Qinba State Key Laboratory of Biological Resources and Ecological Environment (Incubation), Shaanxi University of Technology, Hanzhong 723000, China; 2 Shaanxi Province Key Laboratory of Bio-Resources, School of Biological Science and Engineering, Shaanxi University of Technology, Hanzhong 723000, China; 3 College of Tourism and Aviation Management, Hunan Women’s University, Changsha 410004, China; 4 Hounsfield Facility, School of Biosciences, University of Nottingham, Sutton Bonington Campus, Loughborough LE12 5RD, UK; 5 Technology Innovation Center for Monitoring and Restoration Engineering of Ecological Fragile Zone in Southeast China, Ministry of Natural Resources, Fuzhou 350001, China; 6 Institute of Grain Crops, Xinjiang Academy of Agricultural Sciences, Urumqi 830091, China

**Keywords:** rate of garlic stalk addition, ammonium sulfate, soil nitrate, soil carbon sequestration, soil bacterial community

## Abstract

The lone application of ammonium fertilizer is one of the most commonly used measures to supplement soil nutrients. At the same time, it also causes soil acidification and leads to many environmental problems, such as soil degradation and eutrophication. Garlic (*Allium sativum* L.) stalk (RGS) returning has been widely researched for its benefits related to soil organic carbon (SOC) and crop yields. However, few have researched the effects of the incorporation of RGS mixed with ammonium fertilizer on soil physicochemical properties and the bacterial community composition. We incubated soil with the control (N0); ammonium sulfate (AS); and ammonium sulfate combined with 1%, 2%, 3%, and 5% (rate of the dry soil weight) garlic stalk at 25 °C and 60% water-filled pore spaces (WFPS) for 67 days. We measured the soil properties before and on the last day of the experiment. The results showed that adding RGS increased the contents of soil potassium (K), magnesium (Mg), and total nitrogen (TN), but it significantly decreased soil nitrate (NO_3_^−^). In addition, adding RGS increased the relative abundance of r-strategists and the soil r/K ratio. The α diversity of soil bacteria reached the highest value with 3% treatment. Compared to AS, RGS increased the relative abundance of Firmicutes and Actinobacteria but decreased that of Proteobacteria and Acidobacteria. The function genes of Replication_and_Repair and Cell_Motility were enhanced after adding AS, while the function genes of Metabolism_of_Other_Amino_Acids, Enzyme_Families, and Metabolism were enhanced with increased RGS rates. Although SOC increased, NO_3_^−^ significantly decreased with the increase in the returning levels, which could be due to the strong decreases in nitrifying bacteria with increases in RGS rates from 3% to 5%. Therefore, adding RGS at 3% is suitable for soil bacterial biodiversity and nutrient balance.

## 1. Introduction

Intensive agriculture practices, such as fertilization and tillage, have greatly impacted soil C stocks worldwide. Globally, soils have lost 133 Pg C, mainly due to land-use change [1], tillage, and fertilization practices [2,3]. Similarly, excessive use of ammonium fertilizer causes soil acidification, which is neutralized by SIC, and carbon dioxide is released into the atmosphere, which leads to the loss of SIC [4]. Soil degradation exacerbated by land misuse and soil mismanagement leads to soil C loss [5]. Maintaining or enhancing soil C pools that link with the agricultural ecosystem is significant for global climate change [6].

Agricultural production often involves the return of crop straw to the field. Crop straw is an important source of soil nutrients; for example, the nitrogen and phosphorus accumulated by returning straw to the field in China was equivalent to 13.5% of the total amount of chemical nitrogen fertilizer and 11.5% of chemical phosphorus fertilizer from 2000 to 2017 [7]. Crop straw has been widely recommended for agricultural production in China [8]. About 900 million tons of crop straw is produced every year in China, with an annual growth rate of 2.9% [9]. At present, about 25% of this crop straw is returned to farmland, which has a significant impact on the agricultural ecosystem [9]. Returning crop straw to the field can affect the enzyme activity and microbial community in the soil [10], affects soil N dynamics, and increases the availability of inorganic N in the soil [11,12]. Crop straw decomposition is an important part of the agroecosystem, which can regulate material cycling and affect the soil C pool and nutrient mineralization [13]. For example, returning straw to the field significantly increases the N and SOC content in the topsoil [14,15] and improves the soil K balance and supply capacity [16].

Garlic *(Allium sativum L.)* is one of the important crops that can be used as both a vegetable and a medicine [17]. RGS is a by-product of garlic production, and it is a biological resource [18]. RGS is often returned to the field to maintain soil fertility [17], induce soil biological recovery, and promote nutrient cycling [7]. The decomposition of RGS for 30 or 40 days can promote the growth of wheat (*Triticum aestivum L.*) and lettuce and improve soil enzyme activity [17]. Previous studies on the return of RGS to the field have focused on the effect of RGS on crop yield; for example, a medium addition rate of RGS has the greatest impact on the yield and physiological parameters of eggplant, and an appropriate addition rate of RGS can effectively promote plant growth and regulate the plant reactive oxygen species defense system [19]. In fact, RGS can be used as a natural biofumigant [20]. This is, maybe, because the decomposed RGS contains some allelochemicals and causes allelopathy through releasing the allelochemicals into the environment [21]. In addition, RGS contains high amounts of phytochemicals, including caffeic, p-coumaric, ferulic, and di-ferulic acids, and it has antimicrobial activities against *S. aureus*, *E. coli*, *B. subtilis*, *B. cereus*, and *Proteus vulgaris* [21]. But the effect of RGS on the soil microbial community and the soil physicochemical properties, especially at different rates, is still unclear.

Straw returning provides better conditions for microbial reproduction; for example, adding RGS leads to an increase in soil organic matter (SOM), carbon, and nitrogen availability [17] and increases the number of soil microorganisms to improve soil microbial diversity [22]. Returning sugarcane straw to the field increases the diversity and abundance of soil fungi [23]. Copiotrophs and r-strategists dominate in the early stages when wheat straw is incorporated in soil, and the relative abundance of oligotrophs increases after substrate quantity decline [24]. Previous studies on bacterial life history strategies have revealed that external environmental interference causes the bacterial community to change from K-strategists to r-strategists and gradually recover to K-strategists over time [25]. In the process of karst vegetation restoration, the K/r ratio of soil bacteria is positively correlated with the increase in organic matter [26]. Organic matter accumulation increases the relative abundance of K-strategy bacteria [27]. But a study on returning straw to the field showed that the addition of maize *(Zea mays L.)* straw accelerates the soil nutrient cycle by triggering the growth of microorganisms, especially the fast-growing r-strategists, and synthesizing a large number of enzymes [28]. Returning rice (*Oryza sativa* L.) straw to the field affects the diversity and dynamics succession of the soil fungal community but not the bacterial community [29]. Previous studies have mainly focused on soil fungi because fungi predominate in straw decomposition and fungi can secrete extracellular enzymes to decompose crop straw [23,29]. But many studies have shown that bacterial communities also play an important role in straw decomposition [30,31]. Further studies are needed to investigate the role of soil bacteria in straw decomposition.

We studied the effects of returning RGS to soil at variable rates (1%, 2%, 3%, and 5%) on the soil’s properties, nutrients, and bacterial community composition. Based on previous studies on returning RGS to the field, we proposed three hypotheses. Hypothesis 1: Adding RGS significantly increases the soil C, N, K, Ca, and Mg content. Hypothesis 2: Adding RGS has an effect on the soil bacterial community. Hypothesis 3: A greater proportion of RGS returned to the field is good for maintaining soil fertility, that is, the best proportion is 5% garlic stalk returned to the field. The objectives of this study were to (1) evaluate the impact of RGS incorporation at varying rates (1%, 2%, 3%, and 5%) on soil nutrient dynamics; (2) characterize the effects of RGS amendment on the soil bacterial community composition, diversity, and functional structure; and (3) identify the optimal RGS incorporation rate (1%, 2%, 3%, or 5%) for enhancing soil fertility by analyzing the synergistic relationships between soil properties, nutrient availability, and bacterial community stability.

## 2. Materials and Methods

### 2.1. Preparation of Experimental Materials

The cultivated soil used in the study was collected from a garlic field in Yangling City, Shaanxi Province, China (108°05′ E, 34°18′ N). Garlic had been planted continuously for the past 3 years. Yangling City has a continental monsoon semi-humid climate with four distinct seasons, with precipitation mostly concentrated in July and September. The annual average temperature is 12.9 °C, sunshine hours are 2163.8 h, the annual precipitation is 635.1~663.9 mm, and the frost-free period is 211 days [32]. Soil samples were collected from a depth of 0–20 cm from 12 positions along a zigzag line in the garlic field [33]. The sandy loam soil used in this experiment is classified as anthrosol by the Food and Agriculture Organization (FAO) [34]. We naturally air-dried the soil samples, sieved them with a 2 mm sieve to remove the visible plant roots and stones, and homogenized them for the experiments. The RGS used in the study was collected from the garlic field after the garlic matured. The harvested RGS was air-dried under natural field conditions, then ground into a homogenized powder, and stored in the dark at room temperature according to previous studies [17,19]. The initial properties of the soil and RGS are shown in Table 1.

### 2.2. Experimental Design

We put 300 g of dry soil in sterile glass bottles each and mixed it with distilled water to achieve a soil moisture content of 60% WFPS. The moist soil was pre-incubated for 7 days in the dark at 25 °C to activate soil microorganisms [35]. Afterward, N fertilizer or a mixture of RGS and N fertilizer was added to the glass bottles and mixed thoroughly with the soil. There were 6 treatments in this experiment: control (no fertilization; N0), only ammonium sulfate (AS), and ammonium sulfate with RGS at four rates: 1%, 2%, 3%, and 5% weight of dry soil (AS_RGS_1, AS_RGS_2, AS_RGS_3, and AS_RGS_5). Each treatment was replicated three times. According to the local garlic planting experience, N fertilizer was applied as ammonium sulfate at 0.3 g N kg^−1^ of dry soil in all treatments except N0. Garlic planting in Yangling City is usually in September, and the local average temperature is 22~25 °C [17,18]. Therefore, all treatments were incubated for 67 days at 25 °C and 60% water-filled pore spaces (WFPS). During the whole experiment, the soil moisture was kept constant by the gravimetric method, and the glass bottles were opened for 30 min every 2 days to allow air to circulate. After the experiment, the soil samples were divided into two groups. One group was used to determine the soil properties. The other group of soil samples was used to determine the bacterial community composition. We tested the soil pH, TN, TC, SOC, SIC, MBC, MBN, K, Ca, Mg, Na, NH_4_^+^, NO_3_^−^, and bacterial community composition before and at the end of the experiment.

### 2.3. Physicochemical Analyses

The soil pH was measured by potentiometry according to a soil/liquid ratio of 1:2.5 (*w*/*v*). Soil TC and TN were measured by a high-temperature combustion elemental analyzer (Vario EL MACRO cube, Elementar, Langenselbold, Germany) [36]. SOC was determined by the potassium dichromate external heating method [37]. SIC was measured by a phosphoric acid–solid infrared C sulfur analyzer (solid infrared C flux analyzer: multi EA 4000, Analytik-Jena AG, Jena, Germany). MBC and MBN were measured by the chloroform fumigation–K_2_SO_4_ extraction method. After 24 h of fumigation, both fumigated and unfumigated soils were extracted with 0.5 mol L^−1^ of K_2_SO_4_ (1:4 soil extraction ratio) and centrifuged and filtered. The amount of MBC and MBN was calculated as the difference in the total organic carbon or total N between the fumigated and unfumigated soil samples [38,39]. Soil K, Ca, Na, and Mg contents were measured by ammonium acetate leaching and an inductively coupled plasma emission spectrometer [40]. NO_3_^−^ and NH_4_^+^ were extracted from the soils with KCl solution (1 mol L^−1^) at a solid/liquid ratio of 1:10 (*w*/*v*) and analyzed by a continuous flow analyzer [41].

### 2.4. DNA Extraction and Sequencing

We used the soil DNA kit (PowerMag^TM^ Soil DNA isolation Kit, MO BIO, Carlsbad, CA, USA) to extracted the soil sample DNA in the laboratory. The primers were designed according to the conservative region. The methylcarbamate-degrading (MCD) primers 341F (5′-CCTACGGGNGGCWGCAG-3′) and 805R (5′-GACTACHVGGGTATCTAATCC-3′) were used to amplify the 16S V3-V4 region of bacteria. A sequencing street was added to the end of the primers to conduct PCR amplification and purify, quantify, and homogenize the products, and then the sequencing library was established. After the sequencing library was qualified, we used a NovaSeq 6000 sequencer (Illumina, California, USA) for high-throughput sequencing analysis.

We used FLASH (version 1.2.11, https://ccb.jhu.edu/software/FLASH/ (accessed on 15 July 2024)) to splice the original data [42]. The spliced sequences were filtered by Trimmomatic (version 0.33), and the chimera was removed by UCHIME (version 8.1) to acquire clean tags [43,44]. If they were shorter than 50 bp or contained more than 2 mismatched base pairs or contained ambiguous bases or contained mismatched barcodes or had an average mass score < 20, the raw reads were excluded. At a similarity level of 97%, operational taxonomic units (OTUs) were clustered (Usearch, version 10.0). To ensure the reliability of the results, OTUs with a species relative abundance less than 0.005% were filtered [45]. The taxonomic identity of the representative sequences for each OTU was determined using the Silva reference database (release 132, http://www.arb-silva.de (accessed on 15 July 2024)) for 16S rRNA genes [46]. The classification groups were assigned using the Ribosomal Database Project (RDP) classifier (version 2.2, http://rdp.cme.msu.edu/ (accessed on 15 July 2024)) with a minimum confidence estimate of 80% [47,48]. Alpha diversity indices and relative abundance curves were calculated by Mothur (version 1.30, http://www.mothur.org/ (accessed on 15 July 2024) [49].

### 2.5. Statistical Analyses

We used R (version 4.3.1) and SPSS (version 21) for statistical computation. The data were checked for normal distribution. One-way ANOVA with Tukey’s HSD test was used to test for the significance of the difference in means (statistical significance set at *p* < 0.05). To determine the correlation between the bacterial taxonomic biomarkers of soils with different fertilization practices, the random forest regression method was used to regress the relative abundance of bacteria at the family level. Spearman correlations were used to assess the effect of the RGS-returning rate on the soil bacterial community by comparing their phylum abundance with various soil properties. Principal component analysis (PCA) (based on the abundances of OTUs) was used to examine variability in the soil bacterial community structure under different treatments. We used linear discriminant analysis (LDA) effect size (LEfSe) to compare the differences between different treatments and look for biomarkers with statistical differences. Firstly, the Kruskal–Wallis rank sum test was used to detect all characteristic species, the difference in species abundance among different groups was detected, and significantly different species were obtained. Next, the Wilcoxon rank sum test was used to check whether all subspecies in significantly different species converged to the same classification level. Finally, linear discriminant analysis (LDA) was used to reduce the dimension and evaluate the influence of significantly different species, and a list of different species and the effect size at a specific classification level were obtained [50]. We used PICRUSt software (version 1.1.2) to infer the functional gene composition of samples by comparing the species composition information obtained from 16S sequencing data so as to analyze the functional differences between different samples or groups. Firstly, it is necessary to standardize the generated Feature-table. Next, through the id corresponding to each Feature, the KEGG and COG family information corresponding to the Feature can be obtained so that the abundance of the KEGG and COG family can be calculated, and the Pathway, EC information, and Feature abundance can be obtained from the information in the KEGG database to calculate the abundance of each functional category [51]. We used Sigmaplot (12.0), R Studio, and R (version 4.3.1) for drawing statistical plots.

## 3. Results

### 3.1. Effects of RGS Incorporation on Soil Properties

**Figure 1:**
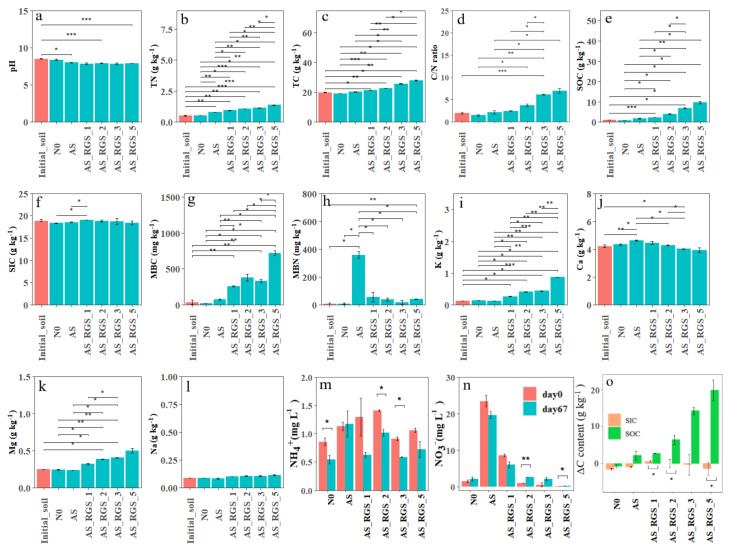
Comparison of the different treatments’ effects on the soil pH (**a**), TN (**b**), TC (**c**), C/N ratio (**d**), SOC (**e**), SIC (**f**), MBC (**g**), MBN (**h**), K (**i**), Ca (**j**), Mg (**k**), Na (**l**), NH_4_^+^ (**m**), NO_3_^−^ (**n**) and C content between days 0 and 67 (∆C) (**o**). The red histograms are the properties of the initial soil at day 0, while the green histograms are the properties of the soils under different treatments at day 67. The orange histograms are the content of SIC, while the emerald-green histograms are the content of SOC. The bars represent standard errors. There were three repetitions for each treatment. Significance: * *p* < 0.05, ** *p* < 0.01, *** *p* < 0.001.

The experiment results revealed significant alterations in the soil physicochemical properties following the addition of AS and RGS. The soil pH exhibited a decline across all treatments, with AS reducing the pH by 5.7% compared to the control (N0), while combined AS_RGS applications caused greater acidification (7.8~8.1% reduction) (Figure 1a). RGS amendments substantially enhanced multiple soil fertility indicators, increasing TN by 87~174% (Figure 1b), TC by 8~40% (Figure 1c), the C/N ratio by 66~376% (Figure 1d), SOC by 133~877% (Figure 1e), MBC by 588~1824% (Figure 1g), and MBN by 159~689% (Figure 1h) relative to N0. These improvements showed dose-dependent responses, with higher RGS application rates yielding greater TC, TN, SOC, K, and Mg concentrations (Figure 1i,k). But Ca exhibited an inverse relationship with RGS dosage, decreasing at higher application rates. Notably, SIC and Na remained unaffected by all treatments (Figure 1f,l). While AS alone increased Ca by 10% (Figure 1j), it showed no significant impact on K and Mg (Figure 1i,k).

AS_RGS initially increased NH_4_^+^ by 6~65% at day 0, with AS_RGS_2 showing the highest concentration (1.4 ± 0.02 mg L^−1^) (Figure 1m). After 67 days, NH_4_^+^ declined by 28~36% in RGS treatments. AS alone maintained stable NH_4_^+^ levels (+4%, *p* > 0.05). NO_3_^−^ responses were more pronounced, with AS inducing a 1486% surge at day 0, while RGS amendments generally reduced NO_3_^−^ by 33~97% (Figure 1n). After 67 days, AS maintained elevated NO_3_^−^ (+826%), whereas AS_RGS_5 showed extreme depletion (−90%) compared to N0.

Control soil lost 26% SOC over 67 days, while AS and RGS treatments increased SOC by 67~226% (Figure 1o). RGS amendments effectively enhanced soil carbon sequestration and nutrient availability.

### 3.2. Effects of RGS on Soil Bacterial Community Diversity

**Figure 2:**
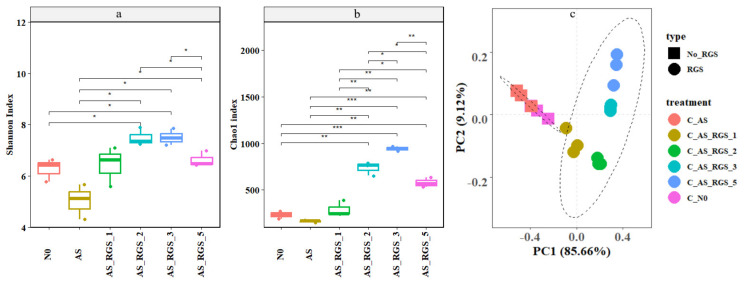
Comparison of the effects of the different treatments on the soil bacterial Shannon index (**a**), Chao1 index (**b**), and bacterial community composition (**c**). No_RGS represents the treatment without RGS (such as control and only added AS), and RGS represents the treatment with RGS. Significance: * *p* < 0.05, ** *p* < 0.01, *** *p* < 0.001. The confidence interval of the ovals is 95%.

AS decreased the Shannon index by 20% and the Chao1 index by 30% compared to N0 (Shannon: 6.3; Chao1: 233.2) (Figure 2a,b). AS_RGS increased the Shannon (3~20%) and Chao1 (25~305%) indexes compared to N0 (Figure 2a,b). The diversity (Shannon index) and richness (Chao1 index) of the soil bacterial community first increased and then decreased with increasing addition rates of RGS, and the peak value appeared in AS_RGS_3 (Shannon: 7.53, Chao1:943.58) (Figure 2a,b).

PCA showed that the bacterial community was not separated in the N0 and AS treatments (Figure 2c). The soil bacterial community with AS_RGS was separated from that with N0 treatment, and the soil bacterial community moved to the upper right with the increase in the addition rate of RGS (Figure 2c).

### 3.3. Effects of RGS on Soil Bacterial Community Composition

**Figure 3:**
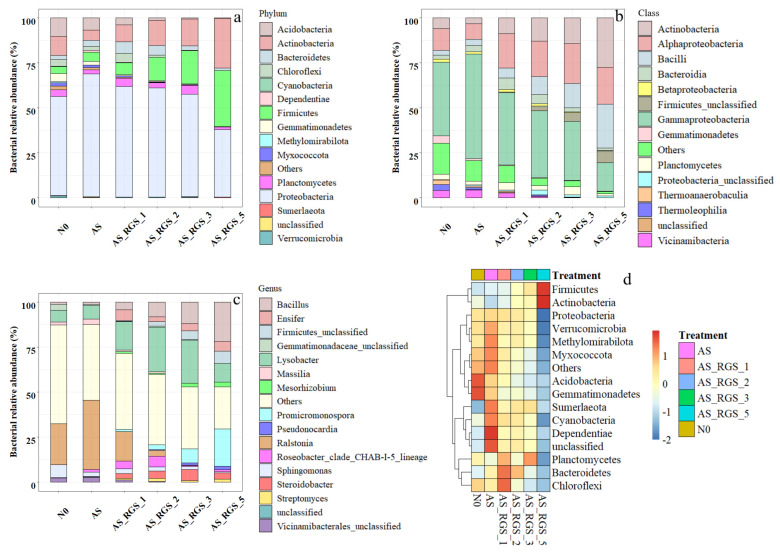
The different treatments’ effects on the soil bacterial community composition at the phylum level (**a**), family level (**b**), and genus level (**c**) at day 67. The heat map shows the effects of different treatments on the relative abundance of bacterial groups at the phylum level (**d**).

PCA indicated that the soil bacterial community structure was similar under AS and N0 treatments but changed by the addition of RGS. The addition of RGS (AS_RGS) not only affected the α diversity of the soil bacterial community but also changed the composition of the soil bacterial community (Figure 3a,d). At the phylum level (Figure 3a), the soil bacteria community under N0 treatment was mainly composed of Proteobacteria (55%), Acidobacteria (10%), Actinobacteria (11%), and Gemmatimonadetes (4%). AS increased the relative abundance of Proteobacteria (68%) and decreased the relative abundance of Actinobacteria (6%), Acidobacteria (7%), and Gemmatimonadetes (2%). However, AS_RGS significantly increased the relative abundance of Firmicutes (7~31%) and Actinobacteria (9~28%) but greatly reduced that of Acidobacteria (4~0%) and Proteobacteria (61~38%) (Figure 3a). The more the addition rate of RGS was increased, the more the relative abundance of Proteobacteria and Acidobacteria decreased, while the more the relative abundance of Firmicutes and Actinobacteria increased (Figure 3a).

The soil bacterial composition at the class level was dominated by Gammaproteobacteria (41%), Alphaproteobacteria (12%), and Actinobacteria (6%) under the control treatment (N0) (Figure 3b). The addition of AS significantly altered this profile, increasing the relative abundance of Gammaproteobacteria to 58% and Bacilli to 4%, while reducing that of Actinobacteria to 3% and Alphaproteobacteria to 8%. In contrast, AS_RGS treatment markedly decreased Gammaproteobacteria (16~40%) and enhanced the proportions of Actinobacteria (9~27%) and Bacilli (6~24%). A clear dose-dependent relationship was observed with RGS application: higher RGS addition rates progressively reduced the relative abundance of Gammaproteobacteria, while promoting increases in Actinobacteria and Bacilli (Figure 3b). RGS amendments counterbalanced the AS-induced dominance of Gammaproteobacteria, favoring taxa associated with organic matter decomposition and nutrient cycling.

At the genus level, the soil bacterial composition under N0 treatment consisted of *Lysobacter* (7%), *Ralstonia* (23%), and *Sphingomonas* (7%) (Figure 3c). The AS treatment significantly increased the relative abundance of *Ralstonia* (39%), while reducing that of *Sphingomonas* (2%) (Figure 3c). Conversely, the AS_RGS treatment substantially decreased the relative abundance of *Ralstonia* (16~0%), with the reduction magnitude showing a positive correlation with the RGS addition ratio. Compared to AS, the AS_RGS treatment enhanced the relative abundances of Bacillus (4~22%) and *Steroidobacter* (3~6%).

### 3.4. Relationship Between Soil Bacterial Community Composition and Soil Physicochemical Properties

**Figure 4:**
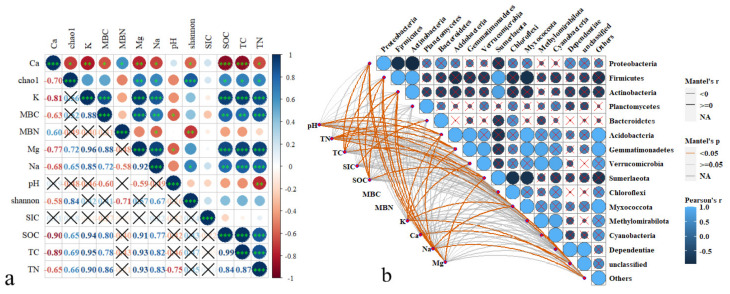
Pearson’s correlation analysis between soil bacterial diversity at the phylum level and soil physicochemical properties, MBC, and MBN (**a**) and Pearson’s correlation analysis between soil bacterial groups and soil biochemical properties, including pH, TN, TC, SIC, SOC, MBC, MBN, K, Ca, Na, and Mg (**b**). Significance: * *p* < 0.05, ** *p* < 0.01, *** *p* < 0.001. TN, total nitrogen; TC, total carbon; SIC, soil inorganic carbon; SOC, soil organic carbon; MBC, microbial biomass carbon; MBN, microbial biomass nitrogen. X indicates that there is no correlation between the two indicators.

Correlation analysis of soil properties and soil bacterial community diversity showed that RGS affected soil bacterial community diversity by acting on soil properties (Figure 4a). The Shannon index was inversely correlated with the soil Ca content and MBN (Figure 4a). The Chao1 index was inversely proportional to the soil Ca content and positively correlated with soil Mg, SOC, TC, and TN (Figure 4a).

The soil pH was positively correlated with the relative abundance of Acidobacteria, Gemmatimonadetes, Verrucomicrobia, and Sumerlaeota (Figure 4b). TN was positively correlated with the relative abundance of Acidobacteria, Gemmatimonadetes, Verrucomicrobia, and Sumerlaeota (Figure 4b). TC was positively correlated with the relative abundance of Proteobacteria, Firmicutes, and Actinobacteria. SIC was positively correlated with the relative abundance of Bacteroidetes. SOC was positively correlated with the relative abundance of Proteobacteria, Sumerlaeota, and Actinobacteria. Soil Na and Mg were also positively correlated with the relative abundance of Proteobacteria, Firmicutes, and Actinobacteria in soil.

There was also a correlation between soil bacterial communities (Figure 4b). Proteobacteria were inversely correlated with Firmicutes and Actinobacteria, while Firmicutes were positively correlated with Actinobacteria and Planctomycetes. Actinobacteria were positively correlated with Sumerlaeota. Acidobacteria were positively correlated with Myxococota and Gammaproteobacteria (Figure 4b).

## 4. Discussion

### 4.1. Effects of RGS on Soil Physicochemical Properties

**Figure 5:**
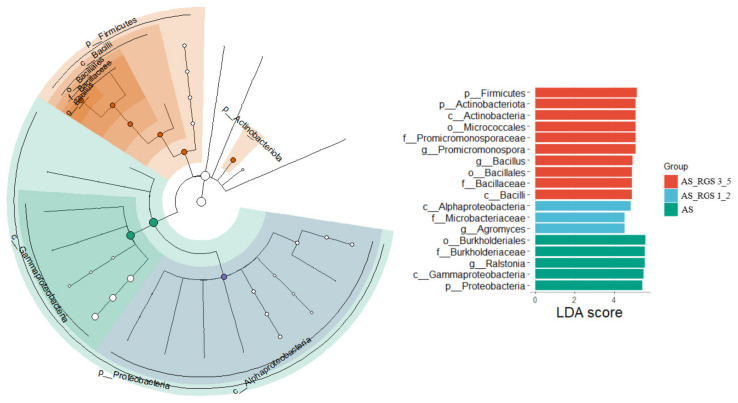
Cladogram using a linear discriminant analysis effect size (LEfSe) method, indicating the significantly different abundant taxa of the bacteria of soils with different addition rates of RGS. LDA scores showed a significant bacterial difference between AS, AS_RGS 1_2, and AS_RGS_3_5 treatments. Green bars represent AS treatment, blue bars represent AS_RGS_1 and AS_RGS_2 treatments, and red bars represent AS_RGS_3 and AS_RGS_5 treatments.

The addition of RGS had no significant effect on soil pH. The addition of RGS increased the soil TC, SOC, and MBC but had no significant effect on SIC. Changes in SIC mainly occur due to soil acidity, but as the pH remained stable after the addition of AS and RGS, it indicates that acidification due to the nitrification of AS at 0.3 g N kg^−1^ was not substantial enough to impact the SIC content in a short time (67 days). RGS is a C-rich organic agricultural waste containing N, P, K, and trace elements that improves the soil nutrient balance and thus can positively impact the C substrate usage rate by soil microbes [11,52]. When the amount of RGS returned to the field increased from 1% to 5%, the SOC increased from 3% to 20%. This could be due to the r-strategists’ (Proteobacteria, Firmicutes, and Bacteroidetes) rapid growth by using the external available C substrate compared to K-strategists, which grow slowly by feeding on SOM [53]. When RGS was added to the soil, soil bacteria (r-strategists 61~89%) used the RGS. Many studies have reported SOC increases after crop straw is returned to the field. For example, Li et al. (2019) reported that SOC increased by 16.4% after straw return [54]. Zhu et al. (2015) also found that with the increase in the straw-returning rate, SOC retention increased, and the straw-returning rate of 50% was the best choice for SOC retention [55]. The content of SOC increased (+0.3~5%) with an increase in the r/k ratio (+4~191 units). The addition of RGS increased the contents of soil K and Mg because the K and Mg of crop straw were easily released and available after straw return [56]. In this study, it was found that although the addition of RGS (AS_RGS) increased soil TN, soil NO_3_^−^ and MBN significantly decreased, and soil NH_4_^+^ was similar to that under N0 and AS treatments. This may be because the decomposition of RGS may promote the use or transformation of NO_3_^−^ by soil microorganisms [57], resulting in the decrease in the NO_3_^−^ content [58]. At the same time, microorganisms may produce some substances that are unfavorable to the soil environment, such as organic acids, which may inhibit the growth and reproduction of microorganisms [58], thus leading to a decline in MBN. The application of AS significantly increased the relative abundance of Gammaproteobacteria compared to adding garlic stalk (Figure 5). Therefore, soil NH_4_^+^ was transformed into NO_3_^−^ through nitrification, and soil NO_3_^−^ increased significantly under AS treatment.

### 4.2. Effects of RGS on Soil Bacterial α Diversity

Recent studies have shown that returning soybean straw to the field affects the relative abundance of soil bacteria [59]. Returning straw to the field increases soil bacterial relative abundance by 1.4 times [60]. Adding wheat straw changes the diversity of soil bacteria rather than fungi [61]. This experiment’s results are consistent with those of these studies, i.e., adding RGS increased the soil bacterial community diversity and richness. This is because the decomposition of RGS provides a large amount of available substances for the growth and reproduction of soil bacteria [62], and the usage rate of soil bacteria on the soil C substrate increases [63]. However, soil bacterial community diversity and richness are not always positively correlated with the amount of RGS returned to the field. This experiment’s results showed that 3% RGS addition (AS_RGS_3) led to the greatest diversity and richness of the soil bacterial community. A possible explanation is that 3% RGS addition is more conducive to the growth and reproduction of microorganisms because a high addition rate of returning straw to the field will block the gas exchange between soil and atmosphere, inhibit the decomposition of straw, prevent the increase in soil bacterial activity and quantity, and reduce the short-term release of straw nutrients [64].

### 4.3. Effects of RGS on the Soil Microbial Community Structure

**Figure 6:**
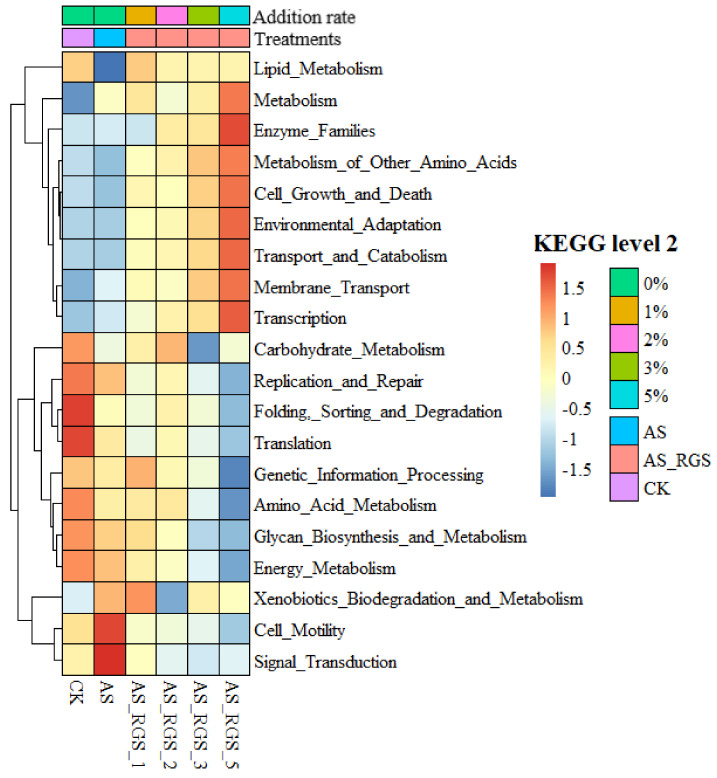
Relative abundances of soil bacterial metabolic genes (KEGG database at level 2) under different treatments. Relative abundance of genes at row normalization by removing the mean (centering) and dividing by the standard deviation (scaling). The colors from blue to red represent the relative abundance of each gene from low to high.

**Figure 7:**
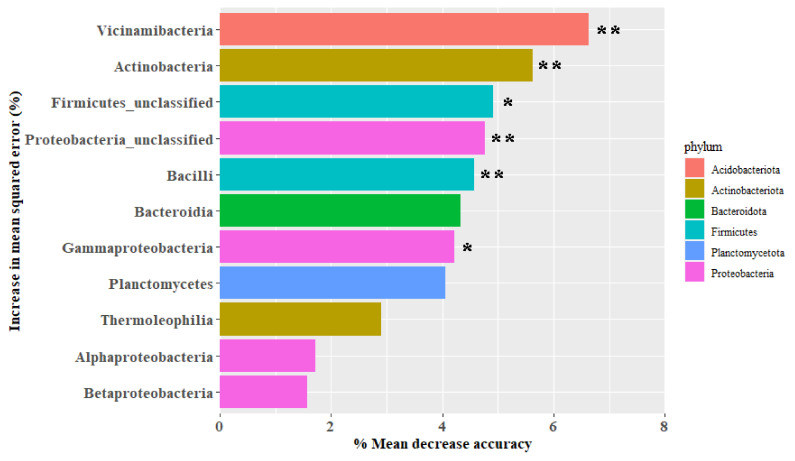
Random forest analysis of soil bacterial community composition under different treatments. The ** symbol denotes values that are significantly different (*p* < 0.01), and the * symbol denotes values that are significantly different (*p* < 0.05).

The addition of AS and RGS changed the soil bacterial community structure. Compared to N0, a single application of AS increased soil N and thus triggered the activity of Proteobacteria [65]. Metabolic function gene prediction showed that the function of Cell_Motility, Signal_Transduction, and Replication_and_Repair was enhanced after adding AS (Figure 6). Proteobacteria are able to fix N [66] and release extracellular enzymes, causing the decomposition of fractions of native SOC [67,68]. The addition of RGS significantly decreased the relative abundance of Proteobacteria (7~24%) and increased the relative abundance of Actinobacteria (4~22%) and Firmicutes (1~26%) compared to AS treatment. Random forest analysis showed that the most important bacteria affecting the bacterial community in all treatments were Acidobacteriota, Actinobacteriota, Firmicutes, and Proteobacteria (Figure 7). In general, applying fertilizer or crop straw can change the soil r/K ratio [69]. This experiment’s results showed that the soil r/K ratio increased with the addition of RGS because r-strategists (61.3%) dominated the bacterial community in N0, and they can use the available substances provided by RGS [70]. Firmicutes can decompose complex organic matter [71]. Therefore, with an increase in the amount of RGS returned to the field, the function genes of Metabolism, Enzyme_Families, and Metabolism_of_Other_Amino_Acids are enhanced (Figure 6).

At the class level, the addition of RGS mainly increased Actinobacteria, Bacilli, and Alphaproteobacteria. Actinobacteria play a crucial role in soil nutrient cycling [72]. Actinobacteria can inhibit pathogens in soil [73] and promote plant growth [74]. Bacilli can promote nutrient cycling between soil and plants [75]. Alphaproteobacteria have a higher C turnover rate [76] and can decompose and use RGS C. Therefore, the addition of RGS can increase the soil-beneficial bacterial groups compared to the application of AS.

## 5. Conclusions

This study revealed that RGS modifies the soil microbial community structure by increasing the r-/K-strategist ratio, creating an ecological niche favoring carbon-retaining bacteria. Incorporating 3% RGS as an organic amendment offers a sustainable strategy to enhance soil fertility, while balancing nutrient dynamics and microbial community. Compared to the conventional lone AS fertilization application, RGS application improves soil health by increasing soil TN, K, and Mg levels, while fostering the beneficial bacterial communities (e.g., Firmicutes, Actinobacteria) critical for SOC stabilization. These findings provide agricultural producers with a science-backed approach to reduce reliance on nitrogen fertilizers, mitigate soil nitrate (NO_3_^−^)-leaching risks, and reduce the greenhouse effect through SOC sequestration.

## Figures and Tables

**Table 1:** Initial properties of soil and RGS in the experiment.

Sample	pH (1:2.5 H_2_O)	TN (g kg^−1^)	TC (g kg^−1^	C/N Ratio	SOC (g kg^−1^)	SIC (g kg^−1^)	MBC (mg kg^−1^)	MBN (mg kg^−1^)	K (g kg^−1^)	Ca (g kg^−1^)	Na (g kg^−1^)	Mg (g kg^−1^)
Soil	8.5	0.5	19.8	2	1	18.8	37.6	7.1	0.1	4.2	0.09	0.3
RGS	7.2	10.8	391.2	36.2	391.2	N/A	N/A	N/A	17.5	5.9	0.5	3.8

Note: TN, total nitrogen; TC, total carbon; SOC, soil organic carbon; SIC, soil inorganic carbon; MBC, microbial biomass carbon; MBN, microbial biomass nitrogen; K, potassium; Ca, calcium; Na, sodium; Mg, magnesium; RGS, garlic stalk; N/A, not applicable.

## Data Availability

Data are contained within the article.
